# Recurrent reversible mutations at *gaf1* driving metastable TORC1 inhibitor resistance in fission yeast

**DOI:** 10.1128/spectrum.01467-26

**Published:** 2026-09-17

**Authors:** Lufeng Dan, Xiaowen Ye, Siyin Liu, Jinping Zhang, Zhihao Qiang, Xingyong Yang, Wenzhu Li

**Affiliations:** 1 Antibiotics Innovation and Resistance Control Key Laboratory of Sichuan Province, School of Pharmacy, Chengdu University 74707, Chengdu, China; 2 Zhejiang Key Laboratory of Molecular Cancer Biology, Life Sciences Institute and The Second Affiliated Hospital, Zhejiang University 12377, Hangzhou, China; Agroscope, Nyon, Switzerland

**Keywords:** metastable inheritance, TORC1 signaling, phenotypic plasticity

## Abstract

Metastable phenotypic inheritance is often attributed to epigenetic mechanisms, but reversible genetic alterations can produce similar instability. Here, we investigated the basis of unstable resistance to TORC1 inhibitor (rapamycin plus caffeine) in *Schizosaccharomyces pombe*. Six independent, metastable resistant mutants were isolated. Genetic mapping positioned the causal lesion to a single Mendelian locus, which sequencing identified as *gaf1*, encoding a GATA transcription factor and a key negative regulator of growth downstream of TORC1. In each mutant, distinct loss-of-function mutations (insertions, deletions, or point mutations) were found in *gaf1* in the resistant state, and these mutations precisely reverted to the wild-type sequence upon loss of resistance. Restoring the wild-type *gaf1* allele abolished resistance, indicating that reversible genetic disruption of *gaf1* is both necessary and sufficient for the metastable phenotype. Furthermore, strong resistance in several strains from a genome-wide deletion library was due to secondary, inactivating mutations in *gaf1*, underscoring its role as a recurrent adaptive target under rapamycin plus caffeine treatment. Mechanistically, *gaf1* inactivation established a distinct basal transcriptome and pronounced derepression of translation and metabolic programs upon drug treatment. While rapamycin plus caffeine triggered extensive chromatin remodeling and H3K9 methylation contributed partially to resistance, these epigenetic changes were most consistent with a downstream modifying layer. Our study shows that metastable drug resistance in fission yeast is predominantly associated with recurrent, reversible genetic inactivation of the central transcriptional regulator *gaf1*, demonstrating how rapidly reversible genetic switches can drive adaptive evolution.

**IMPORTANCE:**

Distinguishing between genetic and epigenetic inheritance is fundamental to understanding how cells adapt to environmental stress. In the fission yeast *Schizosaccharomyces pombe*, rapid and reversible drug resistance is often assumed to be driven by epigenetic switches that change gene activity without altering DNA. However, our study reveals that this instability can be caused by physical mutations in a single gene, *gaf1*, which acts as a genetic toggle. These mutations appear under drug pressure and precisely revert to the original sequence when the drug is removed. We also demonstrate that these spontaneous mutations can contaminate standard laboratory yeast collections, leading to potential misinterpretation of experimental data. These findings broaden our understanding of unstable inheritance and show that DNA sequences can be far more dynamic than previously recognized during rapid evolution and the development of drug resistance.

## INTRODUCTION

Heritable phenotypic variation enables microbial populations to survive fluctuating environments. Among such adaptations, metastable states—those that confer transient advantages yet are reversible—can be selectively beneficial (1–4). The mechanistic basis of such metastable inheritance has often been debated within a framework that pits stable genetic changes against reversible epigenetic regulation (5). However, this dichotomy is increasingly seen as inadequate because the heritable stability of a phenotype is determined more by the molecular features governing its reversibility than by its normal classification as “genetic” or “epigenetic” (4–6). Crucially, certain DNA sequence alterations—including those arising from replication slippage, repeat instability, phase variation, tandem duplication, or microhomology-mediated repair—can themselves function as highly reversible genetic switches (7–9). This blurs the traditional boundary and suggests that rapid genetic change could underlie phenotypes historically attributed to non-genetic mechanisms.

This conceptual ambiguity is particularly salient in studies of antimicrobial and stress-resistance adaptation, where transiently resistant states are often interpreted as epigenetic or stochastic phenotypic switching (10–12). Fungi, for example, can acquire unstable drug resistance through well-characterized epigenetic pathways, such as RNAi-dependent epimutation or heterochromatin-mediated silencing (13–15). However, phenotypically similar states can also stem from rapidly reversible DNA mutations (16, 17), presenting a significant interpretative challenge. Distinguishing between these possibilities requires direct sequence comparisons of resistant lineages and their reverted, sensitive progeny. For microbial populations, the capacity for genetic reversion offers a potential strategy to balance the immediate benefit of resistance against its often-substantial fitness costs (18, 19).

The fission yeast *Schizosaccharomyces pombe* is an ideal system to dissect this problem. Its growth and stress responses are tightly coordinated by the evolutionarily conserved Target of Rapamycin (TOR) pathway (20, 21). Inhibition of TOR complex 1 (TORC1) triggers a starvation-like transcriptional program and growth inhibition (22, 23). While rapamycin alone weakly inhibits wild-type *S. pombe*, its combination with caffeine efficiently suppresses TORC1-dependent growth, providing a robust assay for identifying resistant mechanisms (24–26). This rapamycin-plus-caffeine system is therefore a powerful tool for isolating and characterizing both stable and metastable adaptive states.

A key mediator of the TORC1-dependent response is the GATA-family transcription factor Gaf1. Under nutrient-rich conditions, TORC1 phosphorylates Gaf1, sequestering it in the cytoplasm. Upon TORC1 inhibition, Gaf1 is dephosphorylated, translocates to the nucleus, and executes a transcriptional program that promotes metabolic adaptation while repressing growth-related processes, including tRNA synthesis (22, 27, 28). Consequently, loss of Gaf1 function confers strong resistance to TORC1 inhibition, positioning the *gaf1* locus as a prime candidate for generating adaptive variation under this selective pressure.

Recent work in *S. pombe* showed that unstable rapamycin resistance can arise from rapidly reversible microhomology-mediated tandem duplications at the *ssp1* locus (16). Similar principles have been observed in other microorganisms, including reversible frameshift-associated ethambutol resistance in *Mycobacterium tuberculosis* (17) and reversible chromosomal inversion-mediated switching of antibiotic resistance gene expression in lactococci (29). Together, these examples raise a broader question: how often does reversible genetic mutation, as opposed to purely epigenetic switching, underlie apparent epigenetic inheritance in this system? Addressing this question requires moving beyond single examples to systematically identify and characterize the genetic loci involved. At the same time, the well-established silencing, particularly involving H3K9-methylated heterochromatin, in fungal gene regulation (13, 30) necessitates an integrated understanding of how genetic and epigenetic layers might interact during adaptive evolution.

Here, we report that metastable resistance to rapamycin plus caffeine in *S. pombe* is predominantly associated with recurrent, reversible loss-of-function mutations at a single locus, *gaf1*, in the isolates examined. Through a combination of classical genetics, whole-genome sequencing, and functional genomics, we demonstrate that (i) independently isolated unstable resistant strains carry distinct, disruptive mutations in *gaf1* that revert to wild-type sequence upon loss of resistance; (ii) these mutations are both necessary and sufficient for the phenotype; (iii) *gaf1* emerges as a recurrent adaptive target, as evidenced by secondary inactivating mutations in multiple strains from a genome-wide deletion library; (iv) *gaf1* inactivation installs a distinct transcriptional state that is markedly amplified by rapamycin, directly linking the mutation to the TORC1-inhibited condition; and (v) while H3K9 methylation contributes partially to resistance and rapamycin triggers extensive chromatin remodeling, these epigenetic changes were most consistent with a modifying layer downstream of the primary genetic switch. Our work supports reversible genetic disruption of *gaf1* as a principal mechanism for metastable adaptation, challenging a purely epigenetic interpretation and expanding the mechanistic spectrum of unstable inheritance in response to stress.

## RESULTS

### A genetically reversible, metastable TORC1 inhibitor resistance phenotype maps to a single Mendelian locus

Wild-type *S. pombe* cells plated on rapamycin (100 ng/mL) plus caffeine (10 mM) yielded resistant colonies at a frequency of ∼1 × 10⁻⁵. After propagation in drug-free medium, 14 resistant isolates displayed unstable inheritance, characterized by the progressive emergence of drug-sensitive progeny. Whole-genome and Sanger sequencing confirmed that unstable resistance in eight of these isolates was attributable to reversible mutations in *ssp1*, a gene previously linked to this phenotype (16). The remaining six isolates (designated Epm103–Epm108) were therefore selected for further investigation.

**Fig 1:**
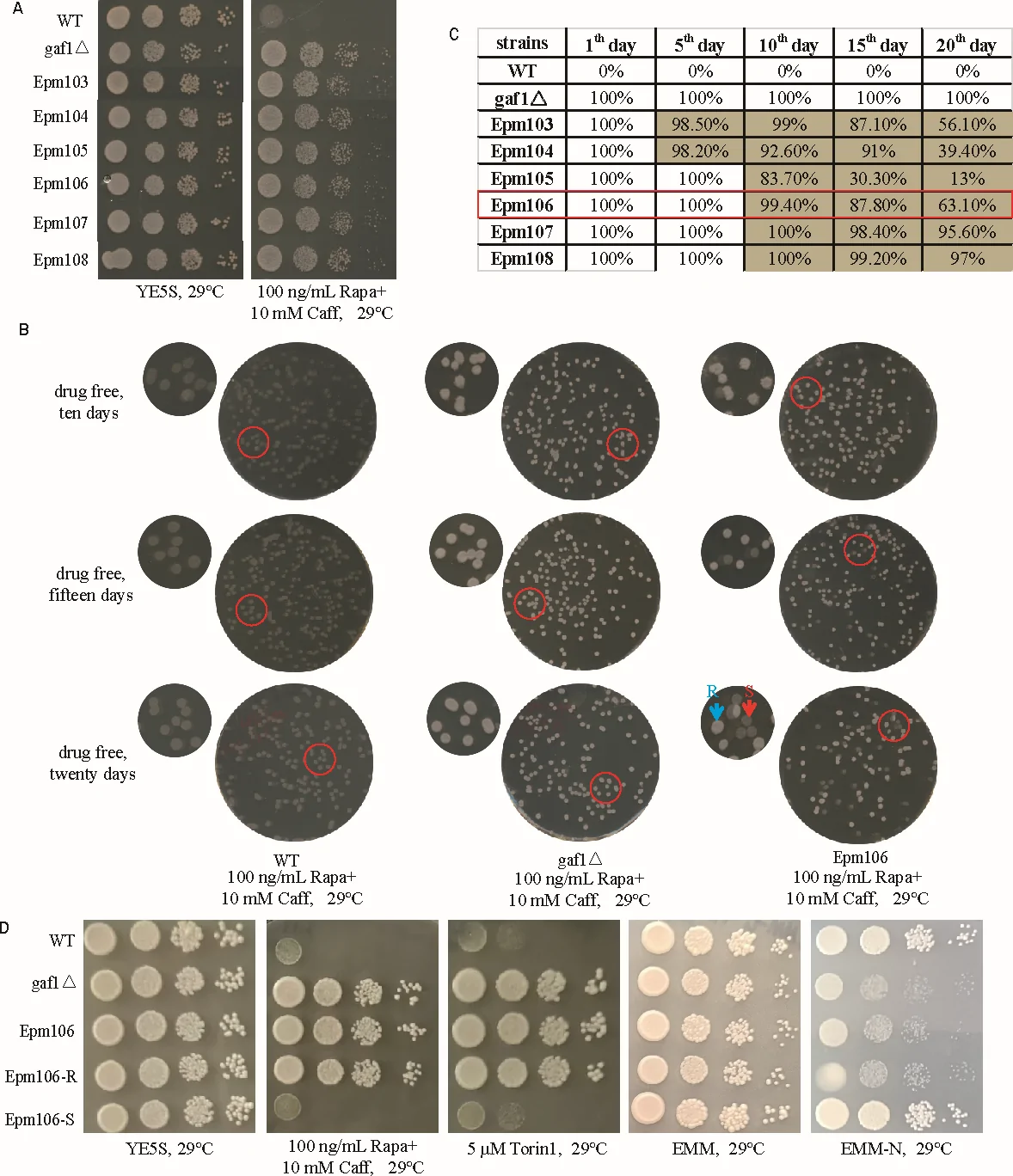
Unstable rapamycin plus caffeine resistance in *S. pombe* and phenotypic reversion of Epm106. (**A**) Serial dilution spot assays comparing the growth of wild-type (WT), *gaf1*Δ, and six unstable resistant isolates (Epm103–Epm108) on YE5S medium with or without rapamycin (100 ng/mL) plus caffeine (10 mM) at 29°C. (**B**) The frequency of resistant progeny decreased progressively during drug-free passage, indicating partial reversal of the resistant state. *gaf1*Δ served as a stable resistant control. Numbers indicate days of culture in drug-free medium before replica plating. Blue arrows indicate resistant colonies, and red arrows indicate sensitive colonies. (**C**) Quantification of the proportion of drug-resistant progeny during serial passage of Epm106 in drug-free medium. (**D**) Serial dilution spot assays of Epm106-derived subclones. Resistant progeny (Epm106-R) and sensitive reverted progeny (Epm106-S) were tested on YE5S, YE5S containing rapamycin plus caffeine, Torin1-containing medium (5 μM), EMM, and nitrogen starvation medium (EMM-N).

The six Epm isolates displayed resistance levels comparable to the *gaf1*Δ control strain in serial dilution assays (Fig. 1A). Among these isolates, Epm106 was selected as a representative strain because its resistance and recovery phenotypes were highly reproducible across independent experiments. Using Epm106, we tracked the change in resistance during serial passage in the absence of drug. The proportion of resistant Epm106 progeny progressively declined, although a substantial fraction of resistant progeny remained after 20 days of drug-free passage (Fig. 1B and C). Importantly, resistant and sensitive descendants could both be recovered from progeny derived from the same resistant clone, supporting stochastic and partial reversibility of the resistance phenotype. Reflecting the genetic metastability of the phenotype, clonally isolated resistant (Epm106-R) and sensitive (Epm106-S) descendants maintained distinct drug-response profiles: Epm106-R retained resistance to rapamycin plus caffeine, whereas Epm106-S reverted to wild-type sensitivity (Fig. 1D).

Next, we asked whether the reversible phenotype was associated with TOR pathway signaling. While Epm106-S restored wild-type sensitivity to the TORC1/2 inhibitor Torin1 (31) and showed a wild-type-like growth response under nitrogen starvation, Epm106-R displayed an altered response under both conditions (Fig. 1D). Together, these data establish that the unstable drug resistance in Epm106 is genetically reversible and coincides with a heritable alteration in TOR pathway activity.

To define the genetic basis of this instability in Epm103–Epm108, each of the six Epm isolates was backcrossed to wild type. Tetrad analysis in all crosses revealed a 2:2 segregation of resistant and sensitive progeny, indicating that resistance is controlled by a single Mendelian locus (Fig. S1A). We then asked whether independent isolates shared the same locus. Intercrosses between the six unstable resistant strains produced tetrads that nearly all showed a 4:0 resistant-to-sensitive segregation pattern on drug plates (Fig. S1B), demonstrating that they are affected at the same resistance-determining locus.

Together, these data show that the metastable rapamycin plus caffeine resistance in the Epm103–Epm108 isolates is associated with recurrent, reversible alteration of a common single genetic locus, which concomitantly perturbs TOR pathway signaling.

### Reversible loss-of-function mutations in *gaf1* are necessary and sufficient for metastable TORC1 inhibitor resistance

**Fig 2:**
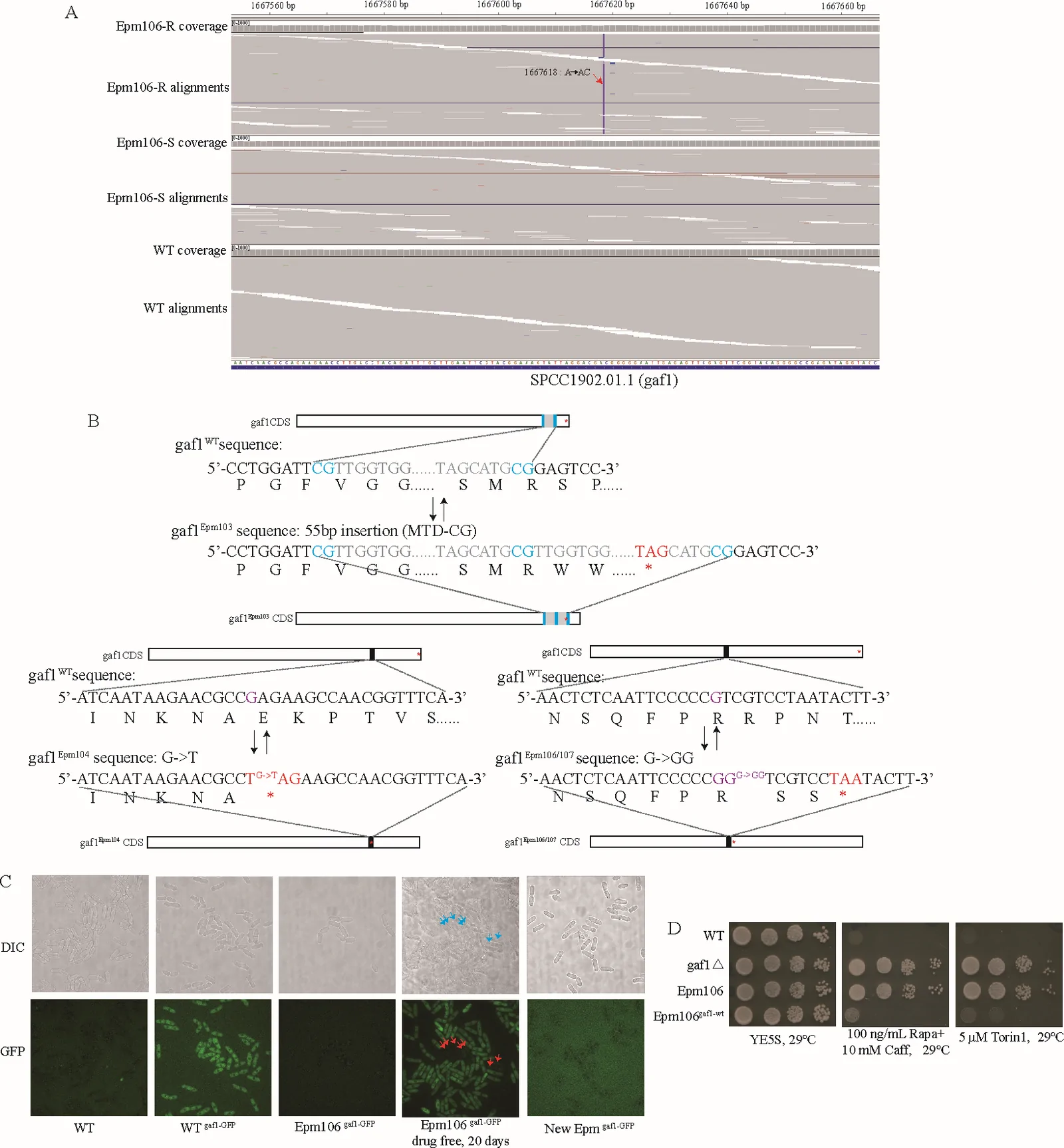
Reversible disruptive mutations at *gaf1* underlie unstable rapamycin plus caffeine resistance. (**A**) Whole-genome sequencing view of the *gaf1* locus in WT, Epm106-R, and Epm106-S. A single-nucleotide insertion at *gaf1* was detected specifically in resistant Epm106-R, whereas Epm106-S restored the wild-type sequence. The arrow indicates the mutant position. (**B**) Sanger sequencing analysis of the *gaf1* coding region in resistant and reverted progeny derived from Epm103–Epm108. Distinct insertions, deletions, or point mutations were detected in resistant progeny, whereas the corresponding reverted sensitive progeny carried the wild-type *gaf1* sequence. Blue letters indicate microhomologous sequences, red letters indicate newly generated stop codons, and red asterisks indicate translational termination. (**C**) Gaf1-GFP fluorescence and corresponding differential interference contrast (DIC) images in WT and representative resistant isolates. Loss of detectable Gaf1-GFP signal was observed in Epm106 and New Epm relative to WT. After 20 days of drug-free passage, both GFP-positive and GFP-negative cells were observed within the Epm106 population. (**D**) Phenotypic rescue after restoration of the wild-type *gaf1* sequence in Epm106. Serial dilution spot assays show that correction of *gaf1* restored drug sensitivity.

To identify the causal genetic lesion, we performed whole-genome sequencing of paired resistant (Epm106-R) and sensitive, reverted (Epm106-S) descendants derived from the metastable isolate Epm106. Comparative analysis revealed a specific insertion mutation within the *gaf1* locus in Epm106-R that was absent in Epm106-S, which carried the wild-type sequence at this position (Fig. 2A). This finding directly linked the unstable resistance phenotype to a reversible DNA sequence alteration in *gaf1*.

We next extended this analysis by Sanger sequencing the *gaf1* locus in all six unstable isolates (Epm103–Epm108). In each case, a distinct disruptive mutation—including point mutations, small insertions, or deletions—was present in the resistant progeny but was reverted to wild-type *gaf1* sequence in the corresponding drug-sensitive revertant (Fig. 2B; Table 1). All identified lesions are predicted to inactivate Gaf1 function, as they introduce frameshifts leading to premature stop codons. Notably, one isolate, Epm103-R, harbored a 55 bp tandem duplication flanked by CG microhomology, consistent with a microhomology-mediated tandem duplication event (16) (Fig. 2B). This observation raises the possibility that replication-associated genome instability may contribute to the generation of reversible *gaf1* alterations.

**TABLE 1:** Summary of disruptive *gaf1* mutations identified in unstable resistant isolates and resistant deletion-library strains^*a*^^,^^*b*^

Strains	Sequence for *gaf1* gene (Sanger)	Sequence for *gaf1* gene (WGS/RNA-seq)
Epm103	55 bp insertion (2195) (MTD)	\
Epm104	G→T (1816)	\
Epm105	C→T (886)	\
Epm106	G insertion (1258)	G insertion (1258)
Epm107	G insertion (1258)	\
Epm108	C deletion (2044)	\
*nbr1*Δ	G deletion (1647)	G deletion (1647)
*pfd1*Δ	C→T (886)	C→T (886)
*ppr1*Δ	T insertion (948)	T insertion (948)

^*a*^ The table summarizes sequence alterations detected at the *gaf1* locus in unstable resistant isolates (Epm103–Epm108) and representative resistant strains derived from the deletion library. Mutation calls obtained by Sanger sequencing and, where available, by whole-genome sequencing (WGS) or RNA-seq are shown. All listed mutations are predicted to disrupt the *gaf1* coding sequence and are associated with the resistant state. In reverted progeny examined from unstable isolates, the wild-type *gaf1* sequence was restored.

^*b*^ “MTD” represents microhomology-mediated tandem duplications. “\” indicates that no data were available.

Having established the correlation between *gaf1* mutations and the resistant state, we next sought to test their functional necessity and sufficiency. First, we examined Gaf1 protein levels. To directly visualize Gaf1 expression, the endogenous *gaf1* gene was C-terminally tagged with GFP in wild-type and Epm106 strains. GFP-tagged Gaf1 was readily detected in wild-type cells but was undetectable in the representative unstable resistant strain Epm106, consistent with the loss-of-function mutations identified in its *gaf1* allele (Fig. 2C). After 20 days of drug-free passage, both GFP-positive and GFP-negative cells were observed within the Epm106^gaf1-GFP^ population, with a substantial proportion of cells regaining detectable GFP fluorescence (Fig. 2C). In parallel, drug-response analysis showed that drug-sensitive descendants emerged during the same passage period, although a proportion of the descendants remained resistant (Fig. S2C).

Second, a genetic linkage analysis showed that the resistance determinant in Epm106 is tightly linked to the *gaf1* locus (Fig. S2B). Third, and most critically, replacement of the mutant *gaf1* allele in Epm106 with the wild-type allele completely abolished drug resistance and restored wild-type drug sensitivity (Fig. 2D).

Taken together, the paired sequence analyses, Gaf1-GFP observations, genetic linkage, and allelic replacement experiments demonstrate that the metastable rapamycin plus caffeine resistance phenotype is associated with recurrent, reversible loss-of-function mutations at the *gaf1* locus. Among these results, the genetic linkage and restoration of drug sensitivity by the wild-type *gaf1* allele establish that disruption of Gaf1 function is necessary and, in the genetic context tested here, sufficient to confer resistance. To determine whether *gaf1* disruption affected basal cellular growth independently of drug treatment, we compared the growth kinetics of Epm106 with WT and *gaf1*Δ control strains under drug-free conditions. Epm106 displayed growth dynamics comparable to both *gaf1*Δ strains and WT cells (Fig. S2A), indicating that the resistance phenotype was not attributable to a general alteration in basal proliferation.

### Secondary mutations in *gaf1* are a recurrent driver of rapamycin plus caffeine resistance in a curated deletion library

**Fig 3:**
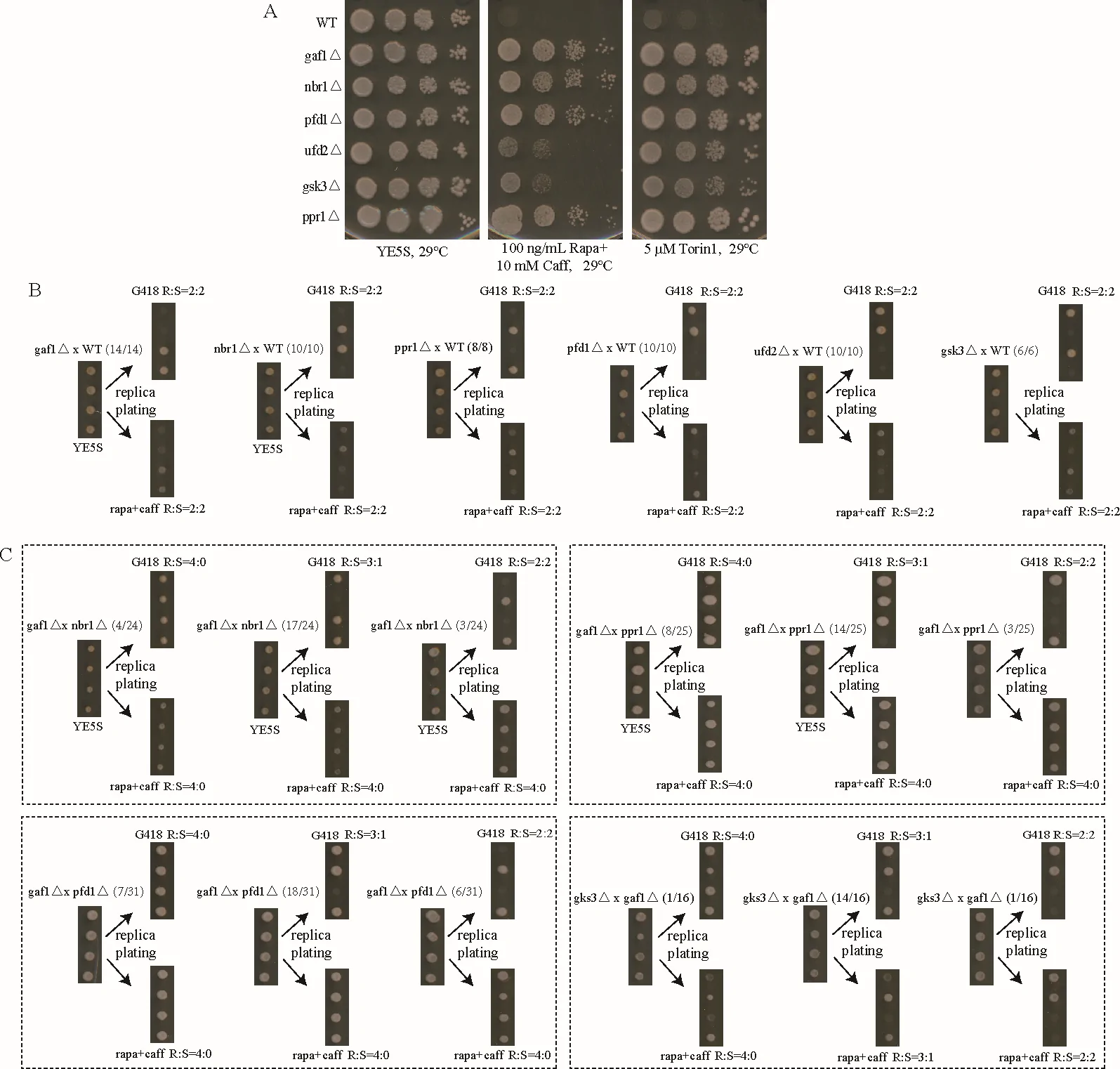
Strongly resistant deletion-library strains genetically map to the *gaf1* locus. (**A**) Serial dilution spot assays evaluating the selected deletion strains recovered from a separately prepared backup copy of the Bioneer version 2.0 *S. pombe* haploid nonessential-gene deletion collection on YE5S medium, YE5S containing rapamycin plus caffeine, and Torin1-containing medium. (**B**) Tetrad analysis after backcrossing resistant deletion-library strains to WT. Progeny were replica plated onto YE5S, G418, and YE5S containing rapamycin plus caffeine. G418 resistance was used to track the KanR-marked deletion cassette. Ratios in parentheses indicate the number of tetrads with the indicated segregation pattern relative to the total number of complete tetrads analyzed. (**C**) Genetic linkage analysis of resistant deletion-library strains by crosses with *gaf1*Δ. For the *gsk3*Δ × *gaf1*Δ cross, rapamycin-plus-caffeine resistance showed variable strength among progeny. In the R:S = 4:0 group, two progeny exhibited weak resistance and two exhibited strong resistance. In the R:S = 3:1 group, two progeny showed weak resistance, one showed strong resistance, and one was sensitive to rapamycin plus caffeine.

Previous large-scale screening of the *S. pombe* gene deletion library had identified 33 strains with reported rapamycin plus caffeine resistance (26). We re-evaluated these strains for resistance to rapamycin plus caffeine and the TORC1/2 inhibitor Torin1. Serial dilution assays confirmed that, while most strains showed weak or modest resistance, a small subset—including *gaf1*Δ—showed strong resistance across version 2 and version 4 deletion collections (Fig. 3A; Fig. S3).

To ensure robustness, we independently restored and re-screened the 33 strains from the version 2 library. This secondary validation confirmed six strains with significant resistance to rapamycin plus caffeine, including *gaf1*Δ, *nbr1*Δ, *pfd1*Δ, *ufd2*Δ, *gsk3*Δ, and *ppr1*Δ (Fig. 3A). Genetic linkage tests between these resistant deletion strains and our *gaf1*-linked Epm106 isolate showed that, except for *gsk3*Δ and *ufd2*Δ, the resistance determinants in the other strains were genetically linked to the Epm106 locus (Fig. S2B). These results point to a common genetic basis for resistance among the remaining deletion-library strains.

**Fig 4:**
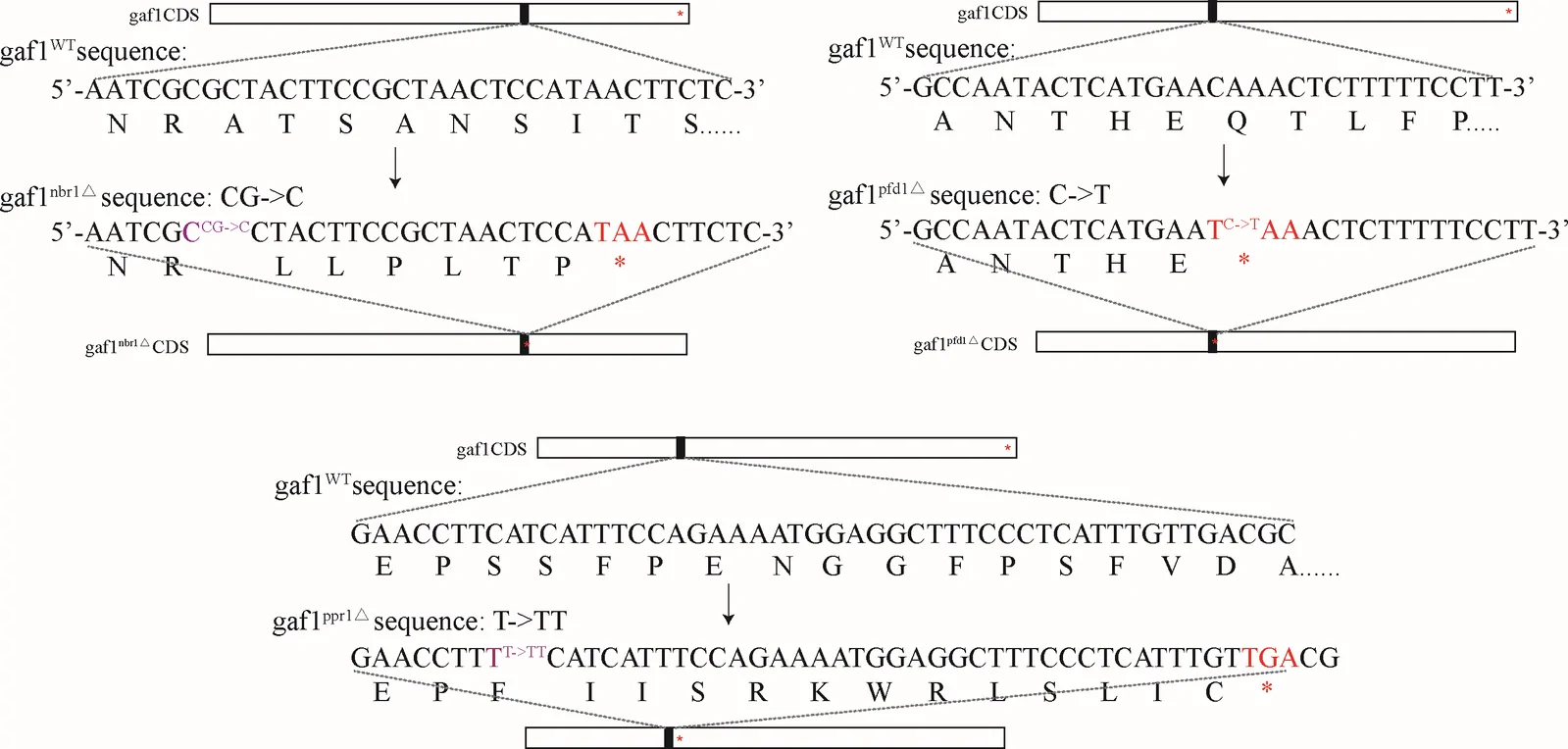
Independent *gaf1* lesions in resistant strains derived from the deletion library. Schematic summary and sequence alignment of the *gaf1* coding sequence in WT and representative resistant deletion-library strains. Distinct mutations identified in these strains are predicted to disrupt the *gaf1* open reading frame and generate loss of function.

A critical question was whether the annotated gene deletions themselves conferred the resistance. We therefore backcrossed each strongly resistant deletion strain to wild type and scored meiotic progeny for cosegregation of rapamycin plus caffeine resistance with the KanR-marked deletion cassette. In strains *nbr1*Δ, *ppr1*Δ, and *pfd1*Δ, the KanR-marked deletion cassette did not cosegregate with drug resistance, indicating that drug resistance was not caused by the annotated deletion (Fig. 3B). Instead, crosses between these strains and *gaf1*Δ confirmed tight genetic linkage to the *gaf1* locus (Fig. 3C). Targeted sequencing of *gaf1* in these strains revealed distinct disruptive mutations (Fig. 4; Table 1), strongly supporting the conclusion that secondary, loss-of-function mutations in *gaf1* were responsible for the strong resistance phenotype. In addition, G418-sensitive but drug-resistant progeny from crosses between *gaf1*Δ and resistant deletion-library *pfd1*Δ strains (designated New Epm) also showed loss of Gaf1 protein (Fig. 2C).

In summary, these results show that strong rapamycin plus caffeine resistance in multiple strains from a widely used deletion library is frequently attributable not to the intended gene knockouts, but to secondary, adaptive mutations that inactivate *gaf1*, underscoring its role as a recurrent adaptive target under this selective pressure.

### Reversible *gaf1* mutations rewire the transcriptional landscape to potentiate rapamycin plus caffeine resistance

Having shown that reversible loss-of-function mutations in *gaf1* are predominantly associated with metastable resistance in the isolates examined, we next sought to understand the molecular basis of this phenotype. A key question is how mutations at this specific locus—making it a recurrent adaptive target—reprogram cellular physiology to confer a fitness advantage under drug pressure.

**Fig 5:**
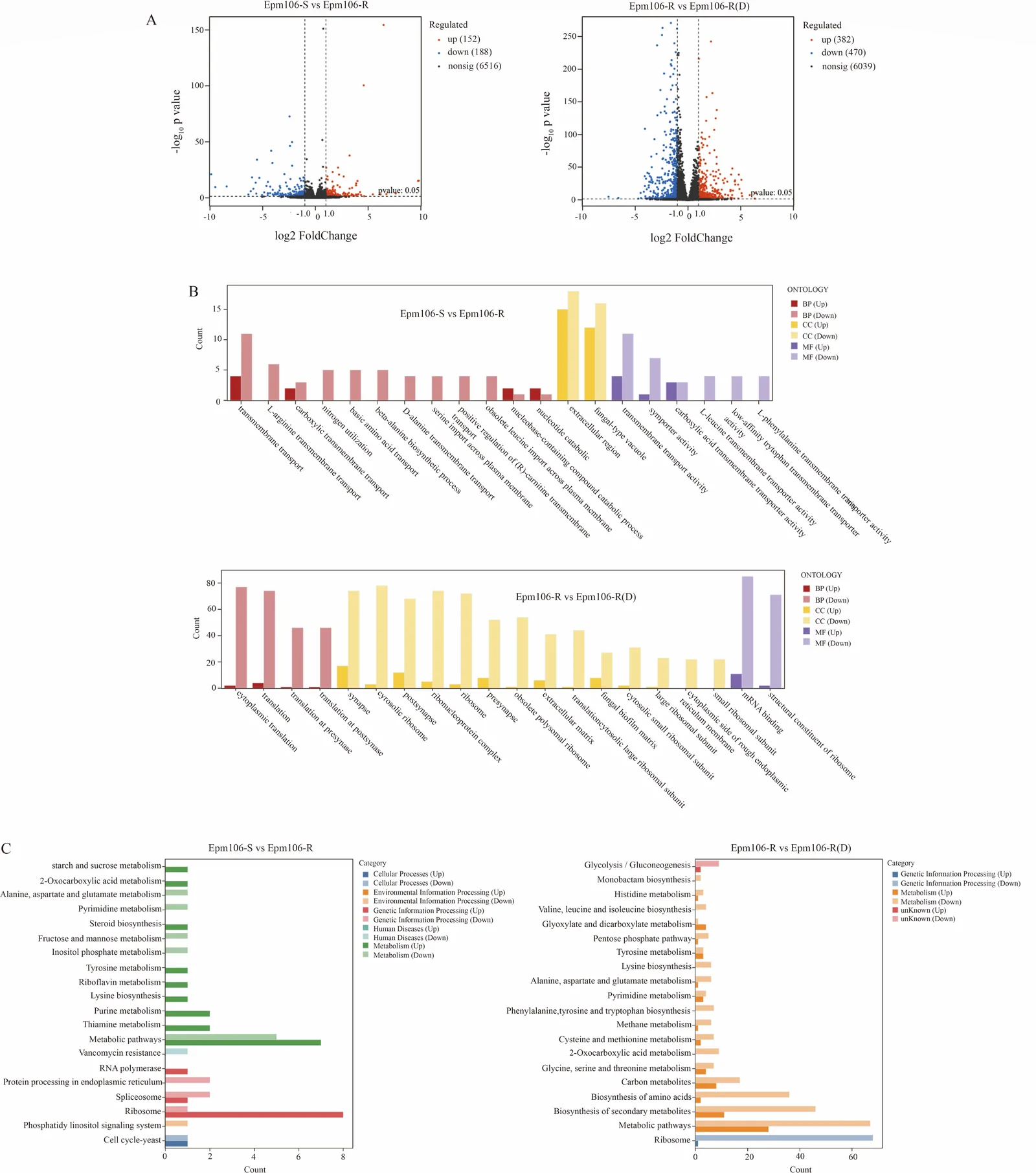
*gaf1* disruption and rapamycin treatment broadly reprogram gene expression. (**A**) Volcano plots showing DEGs. Left, comparison between resistant and reverted progeny derived from Epm106 (Epm106-R versus Epm106-S). Right, comparison between untreated and rapamycin-treated resistant cells (Epm106-R versus Epm106-R[D]). Red and blue dots indicate significantly upregulated and downregulated genes, respectively (adjusted *P* < 0.05), and gray dots indicate non-significant genes. (**B**) Gene Ontology (GO) enrichment analysis of DEGs identified in the comparisons shown in panel **A**. Upper panels, Epm106-R versus Epm106-S; lower panels, Epm106-R versus Epm106-R(D). (**C**) Kyoto Encyclopedia of Genes and Genomes (KEGG) pathway enrichment analysis of DEGs identified in the comparisons shown in panel **A**. Left, Epm106-R versus Epm106-S; right, Epm106-R versus Epm106-R(D).

To directly capture the transcriptional state conferred by the reversible *gaf1* lesion, we performed RNA-seq on isogenic resistant (Epm106-R, carrying the *gaf1* mutation) and sensitive, reverted (Epm106-S, with restored wild-type *gaf1*) progeny. Even in the absence of drug, this comparison revealed 340 differentially expressed genes (DEGs), including 152 upregulated and 188 downregulated genes (Fig. 5A), indicating that the *gaf1* mutation itself installs a distinct basal gene expression program. This program was enriched for metabolic and transport functions, as well as pathways like ribosome biogenesis and phosphatidylinositol signaling (Fig. 5B and C, up). This suggests that *gaf1* inactivation sets a new transcriptional “set point” that may pre-adapt cells to stress.

We then asked how this mutant background responds to the selective agent itself. Rapamycin plus caffeine treatment of the *gaf1*-mutant cells (Epm106-R) elicited a dramatically amplified transcriptional response, with 852 DEGs identified compared to untreated mutant cells, including 382 upregulated and 470 downregulated genes (Fig. 5A). The induced signature was overwhelmingly dominated by terms related to cytoplasmic translation, ribosome function, and core metabolism (Fig. 5B and C, down). This pattern is consistent with a model where loss of Gaf1, a transcriptional repressor of growth-related processes downstream of TORC1, derepresses translation and anabolic programs. Under rapamycin-induced TORC1 inhibition, this derepression in *gaf1* mutants may allow sustained production of proteins essential for growth and survival, thereby conferring resistance. In contrast, wild-type cells, with functional Gaf1, would effectively execute TORC1-mediated repression of these programs, leading to growth arrest.

### H3K9 methylation modulates resistance-associated chromatin remodeling

Having established that reversible *gaf1* mutations drive resistance through transcriptional reprogramming, we sought to explore the potential contribution of chromatin-based regulation, a known mechanism of heritable phenotypic plasticity. We focused on heterochromatin, given its established role in gene silencing and its dependence on H3K9 methylation catalyzed by the sole methyltransferase Clr4 in *S. pombe* (32, 33). Deletion of *clr4* in both the Epm106 (*gaf1* mutant) and *gaf1*Δ backgrounds resulted in a partial reduction of rapamycin plus caffeine resistance, indicating that H3K9 methylation contributes to phenotypic robustness but is not required for establishing the resistant state. Notably, the magnitude of resistance reduction differed between these two genetic backgrounds, suggesting that Clr4-dependent chromatin regulation interacts with the specific adaptive state generated by the underlying *gaf1* lesion (Fig. S4A). In contrast, resistance in the *ssp1^MTD^* control strain, which arises from a different reversible mutation, was largely unaffected by *clr4* deletion, suggesting pathway specificity.

**Fig 6:**
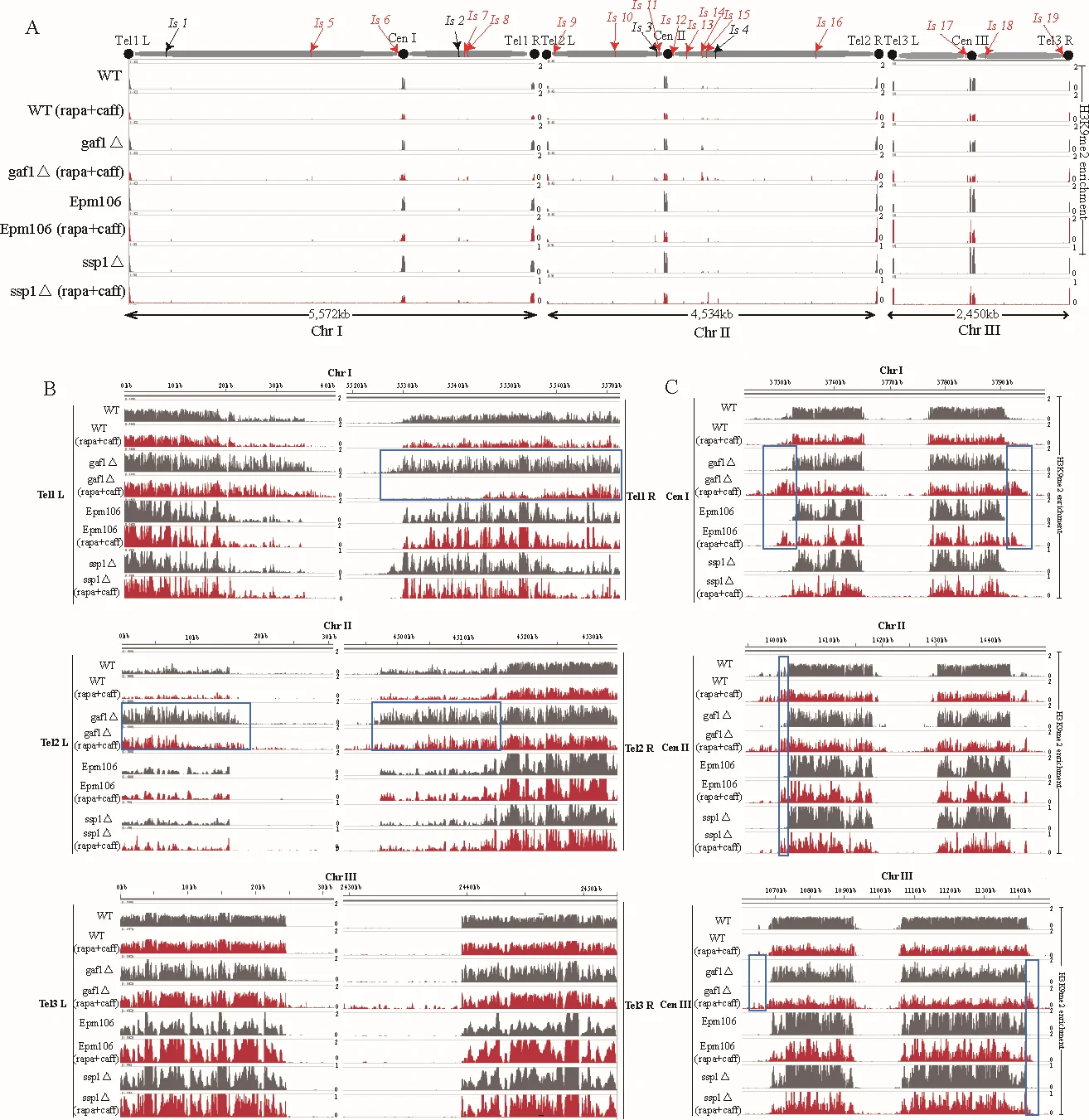
Rapamycin induces extensive remodeling of constitutive and facultative heterochromatin domains. (**A**) Schematic overview of H3K9me2 distribution across chromosomes I-III, indicating constitutive heterochromatin domains and facultative H3K9me2 islands identified in this study. (**B and C**) Genome browser views of H3K9me2 ChIP-seq profiles in WT, Epm106, *gaf1*Δ, and *ssp1* mutant backgrounds with or without rapamycin treatment. Panel **B** shows representative subtelomeric regions, and panel **C** shows representative pericentromeric regions. Blue boxes indicate regions showing notable redistribution of H3K9me2 under rapamycin treatment. For the WT rapamycin plus caffeine (rapa + caff) treatment, cells were exposed to the drugs only during the first 6 h of the experiment.

To gain a genome-wide view of chromatin states associated with resistance, we performed H3K9me2 ChIP-seq in wild-type, Epm106, and *gaf1*Δ strains, with and without rapamycin plus caffeine treatment (Fig. 6A). Rapamycin plus caffeine induced a profound and dynamic reorganization of heterochromatin landscapes in all strains tested. A marked global reduction of H3K9me2 enrichment was observed across canonical pericentromeric heterochromatin domains (Fig. 6C). Concurrently, drug treatment triggered *de novo* spreading of H3K9me2 beyond canonical boundaries at specific loci, most notably at the left boundary of centromere II, a region that appeared particularly sensitive to rapamycin-plus-caffeine-induced chromatin remodeling (Fig. 6B and C).

The pattern and extent of chromatin remodeling, however, varied with genetic background. Under rapamycin treatment, *gaf1*Δ cells showed broad spreading of H3K9me2 across multiple pericentromeric regions, whereas changes in Epm106 were more restricted, primarily evident at centromeres I and II. Additionally, *gaf1*Δ cells displayed reduced H3K9me2 at specific subtelomeric regions (Tel1R, Tel2L, and Tel2R) irrespective of drug treatment (Fig. 6B).

Beyond constitutive heterochromatin, we identified 19 strain- or condition-specific facultative H3K9me2 islands (Fig. S4B). Analysis of these islands revealed distinct patterns of chromatin regulation linked to both genetic background and drug treatment. While H3K9me2 accumulation at meiotic genes (including *mcp7*, *mei4*, and *ssm4*) was observed in most strains regardless of rapamycin plus caffeine exposure, Epm106 exhibited a distinct pattern at these loci, suggesting that the *gaf1* mutant context alters heterochromatin deposition at meiotic regulatory regions. In contrast, rapamycin plus caffeine treatment consistently induced H3K9me2 enrichment at other genomic loci—notably *SPBPB21E7.09*, several tRNA gene clusters, and *SPNCRNA.394*—across nearly all strains, indicating that these sites represent a common chromatin response to TOR pathway inhibition.

Importantly, the landscape of drug-induced facultative heterochromatin was also shaped by genetic background. Distinct patterns of rapamycin-dependent H3K9me2 islands were established in *gaf1*Δ and Epm106 cells. In *gaf1*Δ, prominent enrichment was observed at loci, including *num1*, *ivn1*, *SPBC3H7.08c*, *SPNCRNA.1501*, *rep1*, *rpa12*, *ade6*, and *mug9*, whereas Epm106 displayed differential enrichment patterns at regions, including *avl9*, *vps29*, *iec1*, and *SPNCRNA.394* (Fig. S4). These results demonstrate that the reversible *gaf1* mutation in Epm106 not only phenocopies the constitutive *gaf1*Δ deletion in conferring drug resistance but also shapes a distinct, drug-responsive facultative heterochromatin signature. These differences indicate that chromatin remodeling is influenced by the precise genetic lesion—whether a stable deletion or reversible mutation—and support a model where the *gaf1*-driven adaptive state is reinforced and modulated by context-dependent reprogramming of the facultative heterochromatin landscape. Together, these findings demonstrate that the metastable resistant state, while predominantly associated with reversible *gaf1* mutations in the isolates examined, exists within a context of highly dynamic chromatin architecture. Rapamycin treatment drives extensive remodeling of both constitutive and facultative heterochromatin, with the precise landscape being shaped by the underlying genetic lesion. H3K9 methylation contributes to the phenotypic robustness of resistance, with the chromatin dynamics observed here being most consistent with a modifying layer that operates downstream of the primary genetic switch, rather than as an independent or primary driver in this model of adaptive evolution.

## DISCUSSION

Our study establishes a clear counterexample to the prevailing view that metastable heritable phenotypes are the exclusive domain of epigenetic regulation (13, 14). In *S. pombe*, unstable resistance to TORC1 inhibition is not a result of a purely epigenetic switch but is driven by recurrent, reversible loss-of-function mutations in the GATA transcription factor gene *gaf1*. Our evidence is comprehensive: genetic mapping pinpointed a single locus, whole-genome and Sanger sequencing identified diverse disruptive *gaf1* lesions in resistant strains that reverted to wild-type sequence upon loss of resistance; and—most critically—restoration of the wild-type *gaf1* allele was both necessary and sufficient to abolish the phenotype. This robust causal chain compels a mechanistic reinterpretation of unstable inheritance in this system.

These findings significantly extend the principle that reversible DNA sequence alterations can underpin adaptive phenotypic lability. While a prior study identified microhomology-mediated tandem duplication at *ssp1* as one such genetic switch (16), our work reveals *gaf1* as a second, major locus operating on the same logic. The mutations we recovered—including insertions, deletions, point mutations, and at least a tandem duplication—were structurally heterogeneous but functionally convergent, all leading to Gaf1 loss-of-function. This convergence underscores that selection acts on the phenotypic consequence of transiently inactivating a key regulatory node rather than on a specific sequence alteration, solidifying *gaf1*’s role as a recurrent adaptive target under this selective pressure.

Independent validation of *gaf1*’s central role emerged from our re-analysis of a curated gene deletion library. In multiple strains, strong resistance mapped not to the annotated knockout but to secondary, inactivating mutations in *gaf1*. This result has dual significance: it provides orthogonal support for our genetic model and it serves as a critical caution for functional genomics, highlighting that adaptive phenotypes recovered under selection can be confounded by mutations at highly selectable “hitchhiker” loci like *gaf1*.

Why is *gaf1* such an effective adaptive target? The answer lies in its function as a transcriptional brake within the TORC1 pathway. Gaf1 represses growth-related programs, including ribosome biogenesis and translation, upon TORC1 inhibition (22, 28). Our transcriptomic data provide direct functional validation of this model. Loss of *gaf1* established a distinct basal gene expression state and, under rapamycin treatment, triggered a pronounced derepression of cytoplasmic translation, ribosome biogenesis, and anabolic metabolism. This pattern is broadly consistent with the established role of Gaf1 in coordinating the balance between metabolic and translational programs downstream of TORC1 (28). This establishes a direct, mechanistic link from the genetic lesion (*gaf1* inactivation) to the molecular phenotype (transcriptional reprogramming) and finally to the selective advantage (sustained growth despite TORC1 inhibition). The reversible mutation thus acts as a genetic toggle that flips a pre-wired physiological circuit to a drug-tolerant state.

While establishing the primacy of the genetic lesion, our study also delineates the associated epigenetic landscape, positioning it clearly as a downstream modifier. Deletion of the H3K9 methyltransferase *clr4* partially reduced resistance, and rapamycin treatment induced extensive, dynamic reorganization of both constitutive and facultative heterochromatin. However, several lines of evidence argue against these chromatin changes being the primary driver: the causal sufficiency of the *gaf1* mutation, the distinct chromatin signatures in *gaf1*Δ versus the reversible Epm106 mutant, and the partial phenotype of *clr4*Δ. The chromatin changes are therefore best interpreted as accompanying or modifying features of the resistant state rather than as its principal heritable determinant. This distinction is conceptually important. Chromatin alterations may be prominent and biologically meaningful during adaptive responses; however, their contribution should be interpreted within the context of the initiating genetic state. In our system, the reversible *gaf1* mutation establishes the primary resistant state, whereas Clr4-dependent heterochromatin remodeling influences the degree and robustness of phenotypic expression. The different effects of *clr4* deletion in Epm106 and *gaf1*Δ further indicate that chromatin regulation is not simply proportional to resistance strength, but instead depends on the specific transcriptional state generated by the underlying genetic alteration. The precise targets through which heterochromatin remodeling influences resistance remain to be determined. Our H3K9me2 profiling identified several candidate regions, including tRNA-associated loci and facultative heterochromatin islands near genes involved in developmental regulation and cellular organization. Regulation of tRNA-associated chromatin is particularly intriguing because TORC1 inhibition directly impacts translational control and nutrient-responsive growth programs. In addition, strain-specific H3K9me2 islands suggest that chromatin remodeling may provide a flexible regulatory mechanism for adapting transcriptional outputs to different genetic backgrounds. Future targeted manipulation of these regions will be required to determine their direct contribution to drug resistance.

We therefore propose a hierarchical model: the reversible *gaf1* mutation is the initiating genetic switch that establishes the resistant state; the subsequent transcriptional reprogramming is the core effector mechanism; and the widespread chromatin remodeling represents a downstream, reinforcing layer that may stabilize the new transcriptional program and contribute to the phenotypic robustness. This model resolves the apparent conflict between genetic and epigenetic observations by assigning them ordered, complementary roles.

Our work compels a broader reconsideration of how metastable adaptation is studied. In microbes, fungi, and even cancer, reversible drug-tolerant states are frequently attributed to non-genetic heterogeneity. Our results emphasize that rapidly reversible genetic alterations can generate remarkably similar inheritance patterns and are likely underappreciated in systems where resistant and reverted lineages are not subjected to direct sequencing. Consequently, the key question shifts from “Is it stable or unstable?” to “What is the molecular basis of its heritability and reversibility?” This perspective is particularly relevant for adaptation to intermittent stress, where an ideal solution balances immediate benefit with long-term genetic flexibility.

We acknowledge limitations that point to future directions. Given the diverse mutation types identified in *gaf1*, including insertions, deletions, and point mutations, multiple mutational processes, such as replication errors, replication slippage, or local DNA repair-associated instability, may contribute to the generation of reversible *gaf1* alleles. Replication slippage caused by transient misalignment between template and nascent DNA strands is a well-established mechanism generating reversible insertions and deletions, particularly at structurally unstable genomic regions (34, 35). In addition, DNA repair-associated processes, including microhomology-mediated repair and replication-associated genome instability, can generate diverse sequence alterations that may serve as reversible genetic switches (36).

Although resistance reversal was clearly observed, the persistence of resistant progeny after 20 days indicates that reversal is not synchronous across the population. Longer-term analysis may further define the kinetics of this transition. However, prolonged drug-free cultivation resulted in substantial morphological changes, including flocculent sedimentation, which could introduce additional variability into phenotypic measurements (37). The sequence or structural features that render *gaf1* susceptible to recurrent mutation remain unknown. Our interpretation is that the *gaf1* locus itself functions as a mutational hotspot, rather than its sequence *per se* possessing any specific feature that predisposes it to mutation. Although several identified mutations occurred at or near CG sequences, we consider this pattern likely to be stochastic rather than indicative of sequence-dependent mutagenesis. This view is supported by the structural diversity of the mutations we recovered, which are distributed across the *gaf1* coding sequence rather than clustering at a single site. What makes the *gaf1* locus a hotspot remains unresolved. Possible contributing factors may include its local chromatin environment, replication timing, transcriptional activity, or propensity for DNA secondary structure formation. Defining the molecular basis of this recurrent mutability will require dedicated future investigations, including measurements of locus-specific mutation rates and systematic dissection of the underlying DNA repair and replication pathways. Local DNA sequence context and genome architecture can influence mutation susceptibility by affecting replication fidelity and DNA repair pathway selection (9, 35).

The molecular basis underlying the precise restoration of the wild-type *gaf1* sequence during phenotypic reversion also remains unresolved. Although secondary compensatory mutations that restore the reading frame could theoretically recover Gaf1 function, such events would not necessarily recreate the original nucleotide sequence. The exact sequence restoration observed in the revertants therefore raises the possibility that template-dependent repair, homologous recombination using an intact homologous sequence, or other DNA replication and repair processes—such as synthesis-dependent strand annealing or break-induced replication—may contribute to this reversible genetic switching (38, 39). To explore this possibility, we performed a BLAST search of the *gaf1* genomic region (including both the coding sequence and flanking intronic/intergenic regions) against the *S. pombe* genome to identify potential homologous donor sequences that could serve as templates for precise sequence restoration via homologous recombination. No significant matches outside the *gaf1* locus itself were identified, suggesting that if homologous recombination is involved, it likely utilizes the sister chromatid or the homologous chromosome as a donor rather than an ectopic genomic template. However, we note that our BLAST analysis cannot exclude the possibility of short stretches of microhomology or non-canonical repair mechanisms that fall below the detection threshold of standard homology searches. Neither the involvement of specific repair pathways nor the presence and use of potential homologous donor sequences were directly examined in this study.

Future investigations of local sequence architecture, DNA replication fidelity, potential homologous repair templates, and DNA repair pathways will be required to elucidate the molecular mechanisms underlying both recurrent *gaf1* mutagenesis and its precise reversion. In summary, we have demonstrated that metastable TORC1 inhibitor resistance in *S. pombe* is driven predominantly by reversible genetic disruption of *gaf1*. This work broadens the mechanistic spectrum of unstable inheritance, showing that rapid genetic change can serve as a major engine of adaptive evolution. It provides a framework for distinguishing primary genetic drivers from secondary epigenetic modifiers in complex phenotypes and offers a note of caution for interpreting adaptive screens. Ultimately, it highlights that the boundary between “genetic” and “non-genetic” inheritance is often defined not by the nature of the molecule involved, but by the stability of the alteration it carries.

## MATERIALS AND METHODS

### Strains

The wild-type *S. pombe* strain used in this study was obtained from the laboratory of Xiangwei He at Zhejiang University. Gene deletion strains were obtained from the Bioneer *S. pombe* haploid deletion library (40) or were generated by PCR-based gene targeting using standard procedures (41). The Bioneer collection was used in two physically separate stocks: the original version 2.0 collection and a separately prepared backup copy of version 2.0, as well as version 4.0. The primary screen shown in Fig. S3A was performed with the original version 2.0 stock. The six resistant strains retested in Fig. 3A were recovered from the independent backup copy of version 2.0 and assayed in a separate experiment. The independent screen shown in Fig. S3B was performed with version 4.0. Because these panels derive from different physical stocks and library releases, colony growth among them was not treated as a direct quantitative comparison.

The unstable resistant isolates Epm103–Epm108 and the *ssp1*^MTD^ strain were isolated using a previously described screening strategy (16). The Epm106 *clr4*Δ and *ssp1^MTD^ clr4*Δ strains were generated by replacing the *clr4*^+^ open reading frame with a *kanMX6* resistance cassette via gene targeting. To visualize Gaf1 protein localization and levels, a C-terminal tag was integrated at the endogenous *gaf1* locus in the wild-type and Epm106 backgrounds using a PCR-based tagging strategy. Corresponding transformation-control strains (designated with “-T”) underwent the same procedure with a non-targeting DNA fragment to control for transformation effects. All strains used in this study are listed in Table S1.

### Media and growth conditions

Cells were routinely cultured in yeast extract medium with supplements (YE5S: 0.5% yeast extract, 3% glucose, and appropriate amino acids) at 30°C. For drug selection and assays, rapamycin and caffeine were added to YE5S at final concentrations of 100 ng/mL and 10 mM, respectively. Standard sporulation was performed on malt extract (ME) agar.

### Isolation and stability testing of unstable rapamycin plus caffeine-resistant mutants

To isolate mutants with unstable drug resistance, a single colony of wild-type cells was inoculated into YE5S medium and grown to mid-log phase. Cells were plated onto YE5S agar containing rapamycin (100 ng/mL) and caffeine (10 mM) at a density of 1 × 10^5^ cells per plate and incubated at 29°C for 10 days. Resistant colonies that arose were picked for further analysis.

To assess the stability of the resistance phenotype, each candidate isolate was serially passaged in drug-free YE5S liquid medium at 29°C. Cultures were diluted daily to maintain exponential growth and propagated for up to 20 days. Every 5 days, approximately 200 cells from the culture were spread onto drug-free YE5S plates and incubated for 3 days at 29°C to obtain single colonies. These colonies were then replica-plated onto fresh drug-free plates and plates containing rapamycin plus caffeine, followed by 2 days of incubation at 29°C. Colonies that grew on drug-free YE5S but failed to grow on drug-containing plates were scored as drug-sensitive revertants. The proportion of resistant progeny was tracked over time to quantify the decay of resistance. The stability assay was performed in at least two independent biological replicates for each unstable resistant isolate. The *gaf1*Δ strain, which exhibits stable resistance, was included as a control throughout the experiment.

### Serial dilution growth assays

To quantitatively assess drug sensitivity and resistance, exponentially growing cultures of each strain were harvested and adjusted to a density of 2 × 10^6^ cells/mL in fresh medium. Cells were then subjected to a series of 10-fold dilutions, yielding concentrations from 2 × 10^6^ down to 2 × 10^3^ cells/mL. Aliquots (5 μL) of each dilution were spotted onto YE5S plates with or without drug, as indicated. Control plates (without drug) were incubated at 29°C–30°C for 3 days, while drug-containing plates were incubated for 5 days to allow clear growth discrimination. Plates were subsequently imaged, and growth at each dilution was compared to determine relative resistance levels.

### Genetic analyses and linkage tests

To determine the genetic basis of drug resistance and assess linkage, unstable resistant strains were backcrossed to the wild-type parental strain or crossed to relevant mutant strains on ME medium. After 24–48 h of incubation at 29°C to allow mating and sporulation, tetrads were dissected onto YE5S plates and allowed to germinate for 3 days at 29°C. Resulting colonies were replica-plated onto the following media: (i) YE5S plates, (ii) YE5S plates containing rapamycin (100 ng/mL) plus caffeine (10 mM), and, when appropriate, (iii) YE5S supplemented with G418 (100 μg/mL) to track the inheritance of KanR-marked deletion cassette. Segregation patterns of drug resistance and sensitivity among the meiotic progeny, as well as co-segregation with a genetic marker, were analyzed to test for single-locus inheritance and to map the resistance determinant.

### Fluorescence microscopy

For imaging of Gaf1-GFP, cells were grown to logarithmic phase in YE medium, harvested, and a small aliquot was immobilized on a microscope slide under a coverslip. Live-cell imaging was performed at 29°C using a DeltaVision Elite microscopy system (Applied Precision) equipped with a CoolSnap HQ2 camera (Photometrics). Images were acquired with a 60×/1.4 NA oil objective. For all images, 20 Z-sections were collected, with the vertical distance between optical sections at 0.3 µm. Raw Z-stacking images were deconvolved and combined into maximum intensity projections for analysis using SoftWorx (Applied Precision). For presentation, deconvolved stacks were projected as maximum intensity projections. Final image adjustment for contrast and brightness, and panel assembly, were processed with Photoshop (Adobe).

### Drug treatment for RNA-seq and H3K9me2 ChIP-seq

To compare transcriptional and chromatin states in drug-free versus drug-exposed conditions, we employed distinct treatment regimens for sensitive and resistance strains, as the latter inherently grow in the presence of the drug.

For drug-sensitive strains (wild-type and related controls): cells were first exposed to rapamycin (100 ng/mL) plus caffeine (10 mM) for 6 h to induce an acute TORC1-inhibited state. Following this pulse, cells were harvested, washed, and resuspended in fresh, drug-free medium to allow recovery and continued growth until collection at mid-log phase for subsequent analysis. This “pulse-chase” design aimed to capture sustained molecular changes induced by a transient drug insult.

For drug-resistant strains (*gaf1*∆, Epm106, etc.): as these strains are constitutively resistant, they were cultured continuously in the presence of rapamycin (100 ng/mL) plus caffeine (10 mM) throughout their growth. Cells were harvested at mid-log phase directly from the drug-containing medium.

This differential treatment design should also be described clearly in the corresponding results and figure legends to avoid ambiguity between acute/transient and chronic/continuous drug exposure.

### Whole-genome sequencing

Genomic DNA was extracted using a cetyltrimethylammonium bromide (CTAB)-based protocol. DNA concentration and purity were determined using a NanoDrop2000 Spectrophotometer (Thermo Fisher Scientific). Paired-end DNA libraries with an average insert size of ~350 bp were constructed according to the manufacturer’s specifications and sequenced on an Illumina HiSeq X Ten/Nova Seq platform (Tsingke, Beijing, China) with 150 bp read lengths.

Raw reads were quality-filtered to remove adapters, low-quality bases (Phred score < 20), and reads with >10% ambiguous (“N”) bases. High-quality clean reads were aligned to the *S. pombe* reference genome (ASM294v.2.22) using the MEM algorithm implemented in BWA-MEM2 (v2.2). The average mapping rates were 97.13%, with a mean coverage depth of 572-fold. Mapped reads were sorted, and duplicate reads were removed using Samtools (v1.9).

Single-nucleotide polymorphisms and small insertions/deletions were called using the HaplotypeCaller module in GATK (v4.x). Raw variants were filtered using the following hard filters: QD < 2.0, MQ < 40.0, FS > 60.0, Qual < 30.0, MQrankSum < −12.5, and ReadPosRankSum < −8.0. Additionally, a cluster-based filter was applied (--clusterSize 2 --clusterWindowSize 5).

Functional annotation of variants was performed using SnpEff software based on the reference genome annotation. Variants were categorized as intergenic, upstream/downstream (1 kb), intronic, or exonic. Exonic variants were further classified as synonymous, nonsynonymous, or leading to a premature stop codon (frameshift or nonsense).

### RNA sequencing and differential expression analysis

Total RNA was extracted from yeast cells using a CTAB-based protocol. Briefly, 100–200 mg of yeast cells were collected, washed with DEPC-treated water, and resuspended in 600 µL of preheated (65°C) CTAB extraction buffer containing 2% CTAB, 2% polyvinylpyrrolidone-40, 100 mM Tris-HCl (pH 8.0), 25 mM EDTA, 2.0 M NaCl, and 2% β-mercaptoethanol. The suspension was vigorously disrupted with glass beads using a bead beater for 5 min at 4°C and then incubated at 65°C for 10 min. After the addition of an equal volume of chloroform/isoamyl alcohol (24:1, vol/vol), the mixture was vortexed thoroughly and centrifuged at 12,000 × *g* for 10 min at 4°C. RNA was precipitated from the aqueous phase with an equal volume of ice-cold isopropanol at −20°C for 30 min. RNA was pelleted by centrifugation at 13,600 × *g* for 20 min at 4°C, washed twice with 75% ethanol, air-dried for 5–10 min, and dissolved in 25–100 µL DEPC-treated water. RNA concentration and integrity were assessed using a NanoDrop spectrophotometer and an Agilent 2100 Bioanalyzer (Thermo Fisher Scientific, MA, USA).

For library construction, mRNA was enriched using oligo(dT)-coupled magnetic beads and fragmented into short fragments in fragmentation buffer. First- and second-strand cDNA synthesis, end repair, A-tailing, and adapter ligation were performed according to the manufacturer’s instructions for the Illumina TruSeq RNA Library Prep Kit. Library quality was initially evaluated using an Agilent 2100 Bioanalyzer, followed by duplex-specific nuclease (DSN) treatment. The DSN-treated libraries were further assessed by examining fragment size distribution on the Agilent 2100 Bioanalyzer and quantified by real-time quantitative PCR. Qualified libraries were clustered on a cBot system and sequenced in paired-end mode (2 × 150 bp) on the Illumina NovaSeq X Plus platform (Tsingke, Beijing, China).

Raw sequencing reads were processed with fastp to remove adapters and low-quality bases. Clean reads were then aligned to the *S. pombe* reference genome (ASM294v.2.22) using HISAT2 to generate BAM files for subsequent analyses. Transcripts were assembled and quantified with StringTie, and a merged transcript annotation was generated by comparing assembled transcripts against the reference annotation with gffcompare. Expression levels were normalized to fragments per kilobase of transcript per million mapped reads using the Ballgown workflow based on StringTie-generated input files. Differential expression analysis between groups was performed with DESeq2 using raw read counts as input. Genes with an absolute fold-change > 2 and an adjusted *P* value (FDR) < 0.05 were considered DEGs. These DEGs were subsequently subjected to Gene Ontology (GO) and Kyoto Encyclopedia of Genes and Genomes (KEGG) enrichment analyses.

### H3K9me2 ChIP-seq analysis

Yeast cells were cultured in YES medium at 29°C and harvested at the logarithmic phase. The 10^9^ cells were harvested and digested with Zymolyase 20T at a final concentration of 0.25 mg/mL for almost 1 h at 37°C. The chromatin was pre-warmed to 37°C for 5 min, followed by the addition of micrococcal nuclease (Thermo EN0181) to a final concentration of 240 U/mL for 30 min. The digestion reactions were incubated at 37°C with gentle rolling and were immediately stopped by the addition of EDTA to a final concentration of 2 mM. After centrifugation, the ChIP reaction was performed using anti-H3K9me2 antibody (Abcam, ab1220, 3 μg).

ChIP-seq libraries were constructed using the commercial library preparation kit (KAPA) following the manufacturer’s protocol. Briefly, the DNA libraries were sequenced on the Ion PGM system (Life Technologies). At least 0.5 million reads were obtained for all samples, and all samples showed an overall alignment rate of more than 95%.

H3K9me2 ChIP-seq data were analyzed as described previously (42, 43). Raw reads were assessed using FastQC, followed by adapter trimming and quality filtering with Trim Galore. Raw reads were aligned to the assembly *S. pombe* reference genome (ASM294v.2.22) using Bowtie2 with default parameters. Only high-quality reads with a mapping quality score of ≥30 were retained for downstream analysis, and PCR duplicates were removed using samtools markdup. The resulting alignments in individual samples were normalized by sequencing depth in millions of reads. Peak calling was performed using MACS2 in BAMPE mode, with an effective genome size of 1.2 × 10^7^ and a q-value cutoff of 0.01. The ChIP-seq data were visualized using Integrative Genomics Viewer, and the genome-wide distribution of mapped reads was inspected to verify overall data quality. For comparative visualization, data ranges were adjusted to the same scale or to a twofold range as indicated.

## Data Availability

Strains and plasmids are available upon reasonable request. The whole-genome sequencing (WGS), RNA-seq, and ChIP-seq data sets generated in this study have been deposited in the NCBI BioProject database under accession number PRJNA1513158. All other data supporting the findings of this study are included in the article, figures, tables, and Supplemental Materials.

## SUPPLEMENTAL MATERIAL

Supplemental figures spectrum.01467-26-s0001.pdf Fig. S1 to S4.

Table S1 spectrum.01467-26-s0002.docx Strains used in this study.

ASM does not own the copyrights to Supplemental Material that may be linked to, or accessed through, an article. The authors have granted ASM a non-exclusive, world-wide license to publish the Supplemental Material files. Please contact the corresponding author directly for reuse.
