# Supplementary material for: Recurrent reversible mutations at gaf1 driving metastable TORC1 inhibitor resistance in fission yeast
Source: Microbiol Spectr. 2026 Sep 17;14(10):e01467-26. doi: 10.1128/spectrum.01467-26 (PMC13688802; doi:10.1128/spectrum.01467-26)
Supplement: Supplementary file 1 [file spectrum.01467-26-s0001.pdf]

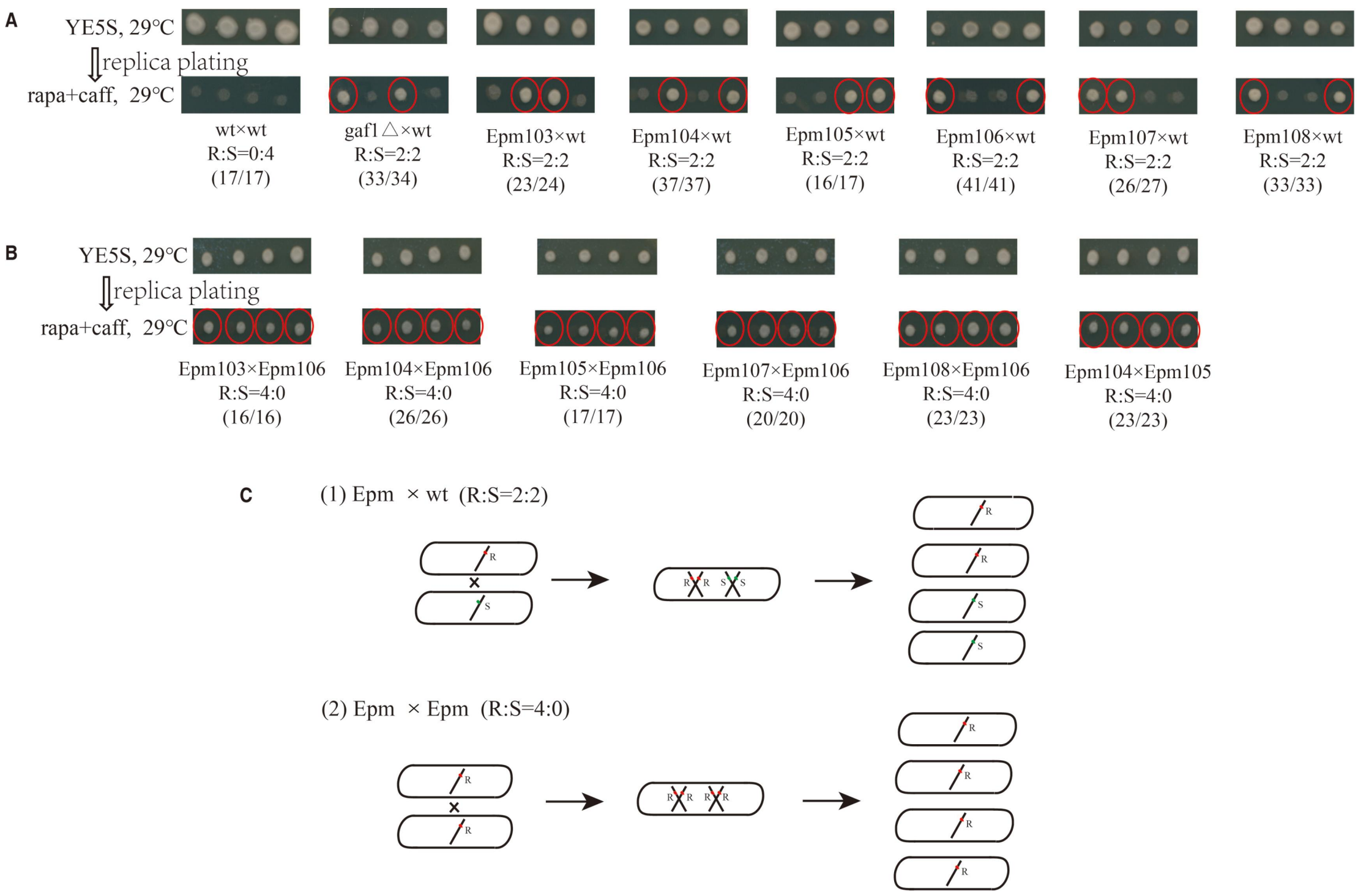

Figure S1. Genetic analyses indicate that unstable resistance in Epm isolates maps to a common single locus.

(A) Tetrad analysis of backcrosses between WT and six unstable resistant strains (Epm103-Epm108). Representative tetrads were replica plated from YE5S onto YE5S containing rapamycin (100 ng/mL) plus caffeine (10 mM) at 29 ° C. Red circles indicate resistant colonies. Segregation ratios of resistant to sensitive progeny are shown below. (B) Genetic linkage analysis by intercrosses among Epm strains. (C) Schematic model of expected segregation patterns. In crosses between Epm and WT strains, a heterozygous mutation yields 2:2 segregation of resistant and sensitive progeny after meiosis. In crosses between Epm strains carrying mutations at the same locus, all progeny inherit resistance, yielding a 4:0 segregation pattern.

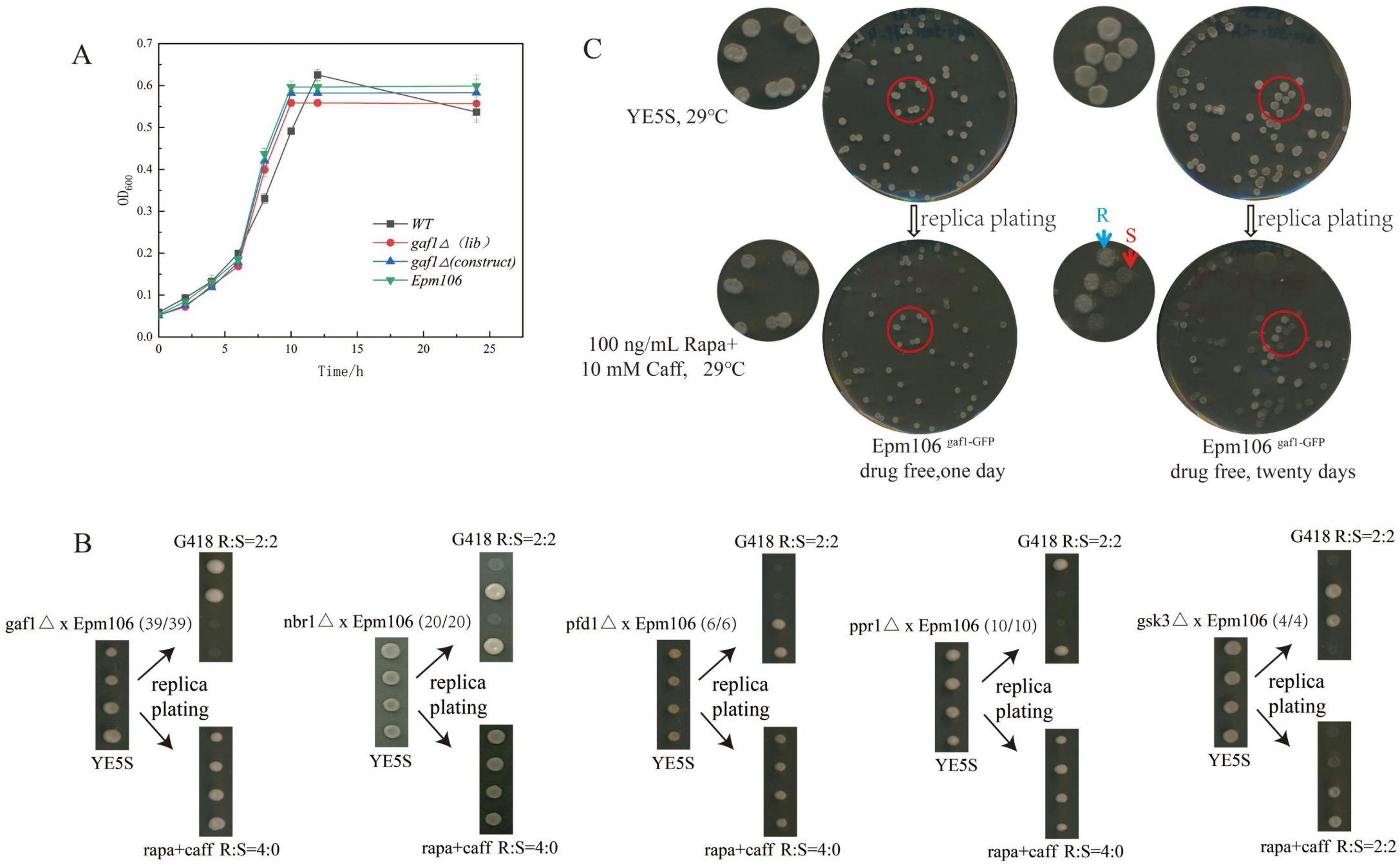

Figure S2. Growth behavior and genetic analyses of Epm106-associated resistance.

(A) Growth curves of WT, library-derived *gaf1 Δ*, independently constructed *gaf1 Δ*, and Epm106 cultured in liquid medium. Optical density at 600 nm (OD600) was measured over time to assess proliferation. (B) Genetic linkage analysis of rapamycin-plus-caffeine resistance by crosses between Epm106 and selected gene deletion strains. Tetrads were dissected and replica plated onto YE5S, G418, and YE5S containing rapamycin (100 ng/mL) plus caffeine (10 mM). The *ufd2 Δ* strain showed a linkage pattern similar to *gsk3 Δ* and was not separately displayed.(C) The frequency of resistant progeny decreased progressively after twenty days drug-free passage for Epm106 $^{gaf1\text{-}GFP}$.

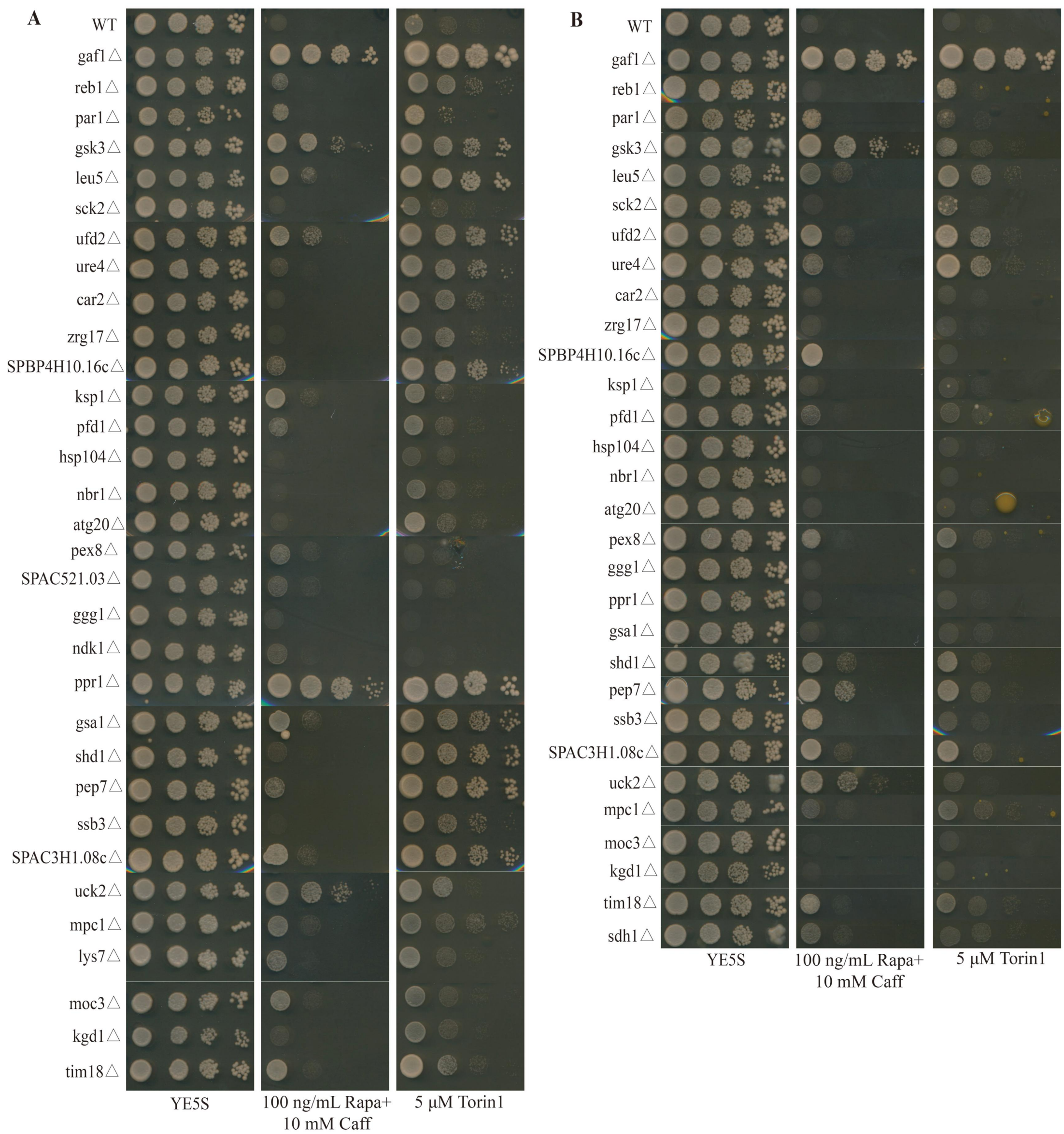

Figure S3. Re-evaluation of rapamycin and Torin1 resistance in deletion-library strains. Serial dilution spot assays of 33 gene deletion strains previously reported to show rapamycin resistance, derived from version 2 (A) and version 4 (B) of the *S. pombe* deletion library. Cells were spotted in 10-fold serial dilutions onto YE5S medium, YE5S containing rapamycin (100 ng/mL) plus caffeine (10 mM), or Torin1（5 μ M）-containing medium and incubated at 29℃. The growth intensities in A and B should not be interpreted as technical replicates or as a direct quantitative comparison.

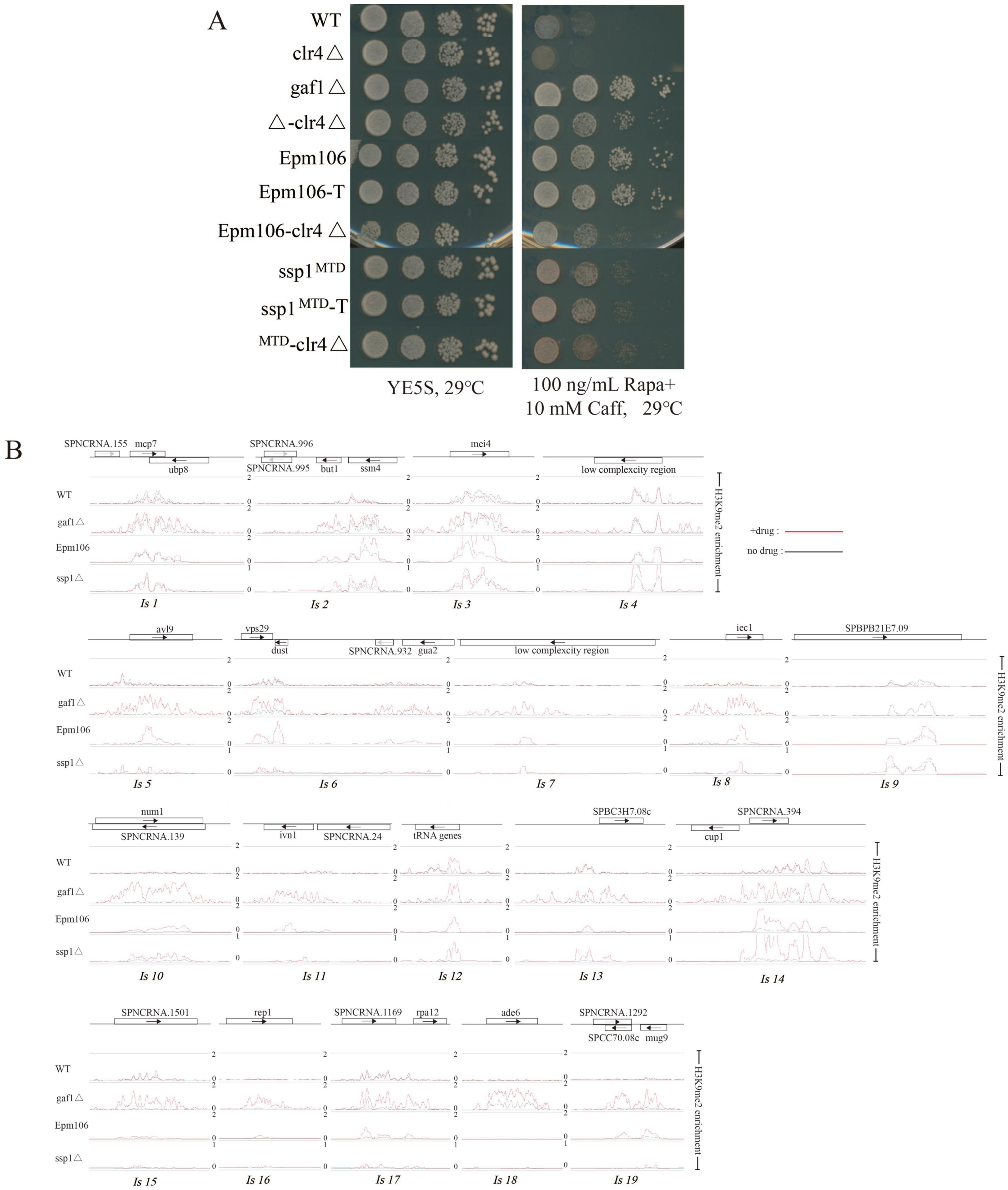

Figure S4. Facultative H3K9me2 islands show strain- and condition-specific changes under rapamycin treatment.

(A) Effect of *clr4* deletion on rapamycin-plus-caffeine resistance in the indicated genetic backgrounds. (B) Genome browser views of H3K9me2 ChIP-seq enrichment across 19 facultative heterochromatin islands (Is1-Is19) in WT, Epm106, *gaf1 Δ*, and *ssp1* mutant backgrounds in the presence or absence of rapamycin. Gene annotations, including protein-coding genes, non-coding RNAs, and low-complexity regions, are shown above the tracks.
