# Supplementary material for: Recurrent reversible mutations at gaf1 driving metastable TORC1 inhibitor resistance in fission yeast
Source: Microbiol Spectr. 2026 Sep 17;14(10):e01467-26. doi: 10.1128/spectrum.01467-26 (PMC13688802; doi:10.1128/spectrum.01467-26)
Supplement: Supplementary file 2 [file spectrum.01467-26-s0002.docx]

**Table S1 Strains used in this study**

| **Name** | **Genotype** | **Source** |
| --- | --- | --- |
| *446* | *h-, ade6-210, leu1-32, ura4D* | He lab |
| *447* | *h+, ade6-216, leu1-32, ura4D* | He lab |
| *Epm103* | *h-, ade-210, leu1-32, ura4, gaf1^MTD^* | Screened in this study |
| *Epm104* | *h+, ade-210, leu1-32, ura4, gaf1^G->T^* | Screened in this study |
| *Epm105* | *h-, ade-210, leu1-32, ura4, gaf1^C->T^* | Screened in this study |
| *Epm106* | *h-, ade-210, leu1-32, ura4, gaf1^G->GG^* | Screened in this study |
| *Epm107* | *h-, ade-210, leu1-32, ura4, gaf1^G->GG^* | Screened in this study |
| *Epm108* | *h-, ade-210, leu1-32, ura4, gaf1^AC->A^* | Screened in this study |
| *Epm106-R* | *h-, ade-210, leu1-32, ura4, gaf1^G->GG^* | Originated from Epm106 |
| *Epm106-S* | *h-, ade-210, leu1-32, ura4, gaf1^WT^* | Originated from Epm106 |
| *WT^gaf1-GFP^* | *h-, gaf1-GFP::kanMX6, leu1-32, ura4D* | constructed in this study |
| *Epm106^gaf1-GFP^* | *h-, gaf1-GFP::kanMX6, leu1-32, ura4D, gaf1^G->GG^* | constructed in this study |
| *New Epm^gaf1-GFP^* | *h+, leu1-32, ura4D, gaf1^C->T^* | *gaf1∆* (lib) cross with *pfd1∆* |
| *New Epm^gaf1-GFP^* | *h+, gaf1-GFP::kanMX6, leu1-32, ura4D,gaf1^C->T^* | constructed in this study |
| *Epm106^gaf1-WT^* | *h-, gaf1::gaf1-kanMX6, leu1-32, ura4D, gaf1^WT^* | constructed in this study |
| *gaf1∆ (lib)* | *h+, gaf1∆::kanMX6, ade6∆::hphMX6, cnt2::ade6-natMX6, leu1-32, ura4D* | deletion library |
| *gaf1∆ (construct)* | *h-, gaf1∆::kanMX6, leu1-32, ura4D* | constructed in this study |
| *nbr1∆* | *h+, nbr1∆::kanMX6, ade6∆::hphMX6, cnt2::ade6-natMX6, leu1-32, ura4D* | deletion library |
| *pfd1∆* | *h+, pfd1∆::kanMX6, ade6∆::hphMX6, cnt2::ade6-natMX6, leu1-32, ura4D* | deletion library |
| *ufd2∆* | *h+, ufd2∆::kanMX6, ade6∆::hphMX6, cnt2::ade6-natMX6, leu1-32, ura4D* | deletion library |
| *gsk3∆* | *h+, gsk3∆::kanMX6, ade6∆::hphMX6, cnt2::ade6-natMX6, leu1-32, ura4D* | deletion library |
| *ppr1∆* | *h+, ppr1∆::kanMX6, ade6∆::hphMX6, cnt2::ade6-natMX6, leu1-32, ura4D* | deletion library |
| *ssp1∆* | *h-, ssp1∆::kanMX6, leu1-32, ura4D* | constructed in this study |
| *clr4∆* | *h-, clr4∆::kanMX6, leu1-32, ura4D* | constructed in this study |
| *gaf1∆-clr4∆* | *h?, gaf1∆::kanMX6, clr4∆::hphMX6, leu1-32, ura4D* | *gaf1∆* cross with *clr4∆* |
| *Epm106-T* | *h-, ade-210, leu1-32, ura4, gaf1^G->GG^* | constructed in this study |
| *Epm106-clr4∆* | *h?, clr∆::kanMX6, ade-210, leu1-32, ura4, gaf1^G->GG^* | constructed in this study |
| *ssp1^MTD^* | *h-, ssp1^MTD-AGCCA^, ade-210, leu1-32, ura4D* | Screened in the previous study(16) |
| *ssp1^MTD^-T* | *h-, ssp1^MTD-AGCCA^, ade-210, leu1-32, ura4D* | constructed in this study |
| *ssp1^MTD^-clr4∆* | *h?, clr∆::kanMX6, ssp1^MTD-AGCCA^, ade-210, leu1-32, ura4D* | constructed in this study |
